# Sexual distress with partnered face-to-face sexual activity: an exploratory qualitative study with heterosexual cis people who seek and do not seek professional help

**DOI:** 10.3389/fpsyg.2025.1553893

**Published:** 2025-08-06

**Authors:** Patrícia M. Pascoal, Gerhard Andersson, Vinicius J. Fischer, Andreia A. Manão, Cátia Oliveira, Catarina F. Raposo, Pedro J. Rosa, Magda Sofia Roberto, Graça Santos, Nuno Tomada, Annamaria Giraldi

**Affiliations:** ^1^Lusófona University, HEI-Lab: Digital Human-Environment Interaction Labs, Lisbon, Portugal; ^2^Clínica Universitária de Psiquiatria e Psicologia Médica, Faculdade de Medicina, Universidade de Lisboa, Lisbon, Portugal; ^3^PSYLAB, Instituto de Saúde Ambiental (ISAMB), Faculdade de Medicina, Universidade de Lisboa, Lisbon, Portugal; ^4^Center for Psychology at the University of Porto, Faculty of Psychology and Educational Sciences of the University of Porto, Porto, Portugal; ^5^Department of Behavioural Sciences and Learning, Department of Biomedical and Clinical Sciences, Linköping University, Linköping, Sweden; ^6^Department of Clinical Neuroscience, Karolinska Institutet, Stockholm, Sweden; ^7^Department of Health, Education and Technology, Luleå University of Technology, Luleå, Sweden; ^8^Centre Hospitalier Neuro-Psychiatrique (CHNP), Rehaklinik, Zentrum fir Psychotherapie, Ettelbruck, Luxembourg; ^9^University of Luxembourg, Student Services, Esch-sur-Alzette, Luxembourg; ^10^Instituto Superior Manuel Teixeira Gomes, Portimão, Portugal; ^11^CICPSI—Centro de Investigação em Ciência Psicológica, Faculdade de Psicologia, Universidade de Lisboa, Lisbon, Portugal; ^12^Hospital da Luz, Odivelas, Portugal; ^13^Instituto de Investigação e Inovação em Saúde, Porto, Portugal; ^14^School of Psychology and Life Sciences (EPCV) of Lusófona University, Lisbon, Portugal; ^15^Sexological Clinic, Mental Health Center Copenhagen, Copenhagen University Hospital, Copenhagen, Denmark; ^16^Department of Clinical Medicine, Faculty of Health and Medical Sciences, University of Copenhagen, Copenhagen, Denmark

**Keywords:** sexual distress, sexual pleasure, sexual dysfunction, partnered sexual activity, help-seeking, clinical practice, qualitative approach, sexual problems

## Abstract

**Introduction:**

Sexual distress is interrelated with mental health and relationship quality and is fundamental for establishing a diagnosis of sexual dysfunction, even though it also affects people who do not seek professional clinical help. Research on sexual distress related to partnered sexual activity is limited, and no comprehensive model exists to guide research or clinical interventions. We conducted an online cross-sectional qualitative study to: 1) explore the reasons why people experiencing sexual distress in partnered face-to-face sexual activity do not seek professional clinical help; 2) analyze the experiences of participants’ of sexual distress in partnered sexual activity; 3) reflexively compare the experiences reported by participants who seek and do not seek professional help; and 4) reflexively compare experiences across genders.

**Methods:**

We performed reflexive thematic analysis on 438 heterosexual people answers (*M*_age_ = 41.06, *SD* = 12.19), including 306 women (69.7%) and 132 men (30.1%).

**Results:**

Most participants (54.1%) had not sought professional clinical help but wanted to do so. Some participants (13.2%) expressed a desire for clinical consultations but reported financial or time constraints. Using the reflexive thematic analysis on the qualitative data provided, we created three themes: (1) Sexual (dys)function (It’s the function), which focuses on sexual function and lack of pleasure; (2) Intimacy dynamics (It’s us!), which discusses relationship challenges; (3) Intrapersonal struggles (It’s me!), which highlight individual factors, some influenced by social messages. Comparison across groups revealed that people who sought professional clinical help emphasise genital function and negative emotions, and women highlighted experiencing sexual pain, while men emphasised desire discrepancies and erectile disorder.

**Discussion:**

Our results demonstrate that difficulties related to sexual pleasure and with penetrative sex are important sources of distress in partnered sexual activity, which is in line with DSM and ICD frameworks of sexual dysfunction. Participants’ accounts show that pre-existing psychological characteristics, partnered communication, cognitive, and emotional factors are key factors to shape the experience of sexual distress related to sexual dysfunctions. This has implications for clinical work as interventions should target transdiagnostic individual factors that may not be sexual specific (e.g., repetitive negative thinking) as well as couple-level factors (e.g., communication). Internet-based integrative therapies directed at these factors may be a promising venue for those who experience sexual distress with partnered sexual activity and are reluctant to seek in-person sexual healthcare.

## 1 Introduction

According to DSM-5-TR (American Psychiatric Association, 2022) and ICD-11 (World Health Organization, 2019), the experience of some level of sexual distress is an essential condition to establish a diagnosis of sexual dysfunction, and different researchers and clinicians have advocated for its essential role in the experience of sexual dysfunction (Hendrickx et al., 2013). Distress related to face-to-face partnered sexual activity is essentially linked to problems with sexual function (Zheng et al., 2020) and, despite its crucial significance in clinical settings, can also be found in community samples (Zheng et al., 2020). In both contexts (i.e., clinical settings, community samples), the experience of sexual distress related to sexual function in the context of face-to-face partnered sexual activity is associated with poorer mental health (e.g., Ventus et al., 2017) and poorer relationship quality (e.g., Nickull et al., 2022), which establishes it as an important correlate of people’s overall health. Understanding the nuances of sexual distress, namely distress related to face-to-face partnered sexual activity and why people do not seek health services contributes to the Sustainable Development Goals of the United Nations (United Nations, 2015), specifically to Goal 3: Good Health and Well-Being For All.

Research developed worldwide consistently demonstrates that not everyone experiencing distressing sexual problems actively seeks or receives clinical professional help. In a study developed in Europe, America, Africa, Asia, and Australia, data were collected in 31 countries (Moreira et al., 2005) and found that among those who experienced a sexual problem, only 18.0% of men and 18.8% of women had sought medical help. According to Moreira et al. (2005), the reasons for participants not seeking professional help were some beliefs, such as considering sexual problems a normal part of ageing, doubting a doctor’s ability to help, or lacking the confidence to discuss these issues with healthcare providers. Other research, meanwhile, developed in France (Buvat et al., 2009), the United States (Laumann et al., 2009), Belgium (Hendrickx et al., 2016a, 2016b) and Canada (Lafortune et al., 2023) report similar data, confirming most of those with sexual problems reported to be distressed. Still, most of them did not seek professional clinical help. While the reasons for avoiding professional help may vary (e.g., low level of distress, long waiting lists), many individuals experience distressing sexual problems without having clinical support. Considering this data, a comprehensive approach to sexual distress needs to include reports and knowledge derived from people in clinical settings (patients and professionals) but also from those who are not receiving professional help, receiving professional help and nonetheless experience distress and distress and may be compatible with or at risk for a formal clinical diagnosis but remain understudied.

Sexual distress is an umbrella concept and has been defined in multiple ways. For example, Witting et al. (2008, p. 5288) state that sexual distress is “characterized by negative feelings and anxiety about one’s sexuality or sexual activities” and Hendrickx et al. (2016a, 2016b, p. 1662) define it as “distress that is experienced due to a sexual impairment.” Furthermore, sexual distress has been assessed using multiple measures that are aligned with different visions of the concept. For example, the *Sexual Satisfaction Scale for Women* (SSS-W, Meston and Trapnell, 2005) describes sexual distress as distress about sexual self-concerns, and the widely used *Sexual Distress Scale-Revised* (SDS-R, Derogatis et al., 2008; Santos-Iglesias and Walker, 2018) describes it as sexuality related personal distress (e.g., distress about sex life). Some other studies that measure sexual distress do not use a scale and instead ask questions such as “During the past 4 weeks, how much distress or worry has your own sexuality caused you?” (Bancroft et al., 2003) or “For each type of impairment reported, women can indicate whether impairment is causing them personal, perceived partner, and interpersonal distress” (Hendrickx et al., 2016a, 2016b). This diversity reveals the various perspectives that researchers can take and reveals the inherent difficulties with providing a basis for comparison across studies, as well as developing an empirical ground to sustain a comprehensive model of sexual distress, namely of sexual distress in partnered face-to-face sexual activity.

Recent research using the SDS-R has demonstrated that sexual distress and psychological distress are statistically significantly associated, but not to the extent that may be considered equivalent dimensions (Raposo et al., 2023; Raposo et al., 2025). The proximity among these two concepts (psychological distress and sexual distress) may be the reason why sexual dysfunctions, characterized by the experience of sexual distress, can be included in the internalizing spectrum of psychopathology (Forbes and Schniering, 2013; Forbes et al., 2016) which is in line with a dimensional approach to psychopathology that looks at common explanatory processes across disorders (Kotov et al., 2017). This view is corroborated by recent research that has found that emotional problems (e.g., depression; Ventus et al., 2017), transdiagnostic cognitive processes (e.g., worry; Pascoal et al., 2020) and difficulties with emotion regulation (Raposo et al., 2023) are related to sexual distress. However, due to the widespread use of the SDS-R, which adopts a generalist approach to measuring sexual distress, it remains unclear whether the identified transdiagnostic factors are specifically linked to sexual activity or to other types of sexual problems that may be a source of distress, such as discomfort with one’s sexual identity, which are not directly related to sexual activity.

The difficulties with describing with precision what sexual distress is have already been presented by diverse researchers (e.g., Hendrickx et al., 2013). Between 2008 and 2009, a project about how laypeople conceptualize distressing sexual problems developed in Portugal produced some preliminary data from different qualitative studies (Pascoal et al., 2008; Pascoal et al., 2009). These qualitative studies highlighted that people defined distressful sexual problems as problems with sexual function (e.g., “not having a good erection”), dissatisfaction with sexual life (e.g., “not having the type of sex I would like”), partner problems related to sexuality (e.g., “selfish partners that only want to reach orgasm and please themselves”), problems related to sexual orientation (e.g., “I find it difficult to find a place in the gay scene”) or gender identity (e.g., “I feel I live a lie, I wish I were a man”) or problems related to the preference for sexual practices (e.g., “my partner is not into kink, but I am”). Even though this exploratory preliminary set of studies was promising because they highlighted that sexual problems should be considered outside a focus on genital performance, their results were insufficient as they reflected a broad conception of sexual distress and did not address the specificity of sexual distress with partnered face-to-face sexual activity.

More recently, a study with clinical sexologists exploring how professionals approach sexual distress related to sexual function (SDRSF) (Raposo et al., 2024) showed that SDRSF is explained by problematic sexual function, as expected, but this is interrelated with individual emotional (e.g., emotional disorders) and cognitive (e.g., internalized sexual stigma) processes, as well as interpersonal (e.g., communication difficulties) and societal (e.g., media pressure) processes. This study focused specifically on distress related to sexual function and did not aim to address distress related to overall sexual activity. However, the results revealed though clinician’s answers moved beyond a strict focus on sexual function, suggesting a broader perspective to be taken. It highlighted that when approaching SDSRF a comprehensive approach that looks at factors involved in sexual distress related to partnered face-to-face sexual activity as a whole moving beyond a strict genital focus.

Despite its clinical relevance, research about sexual distress with partnered face-to-face sexual activity has been scarce, and there is no comprehensive model to understand it and, subsequently, guide research or clinical intervention. Previous research has explored sexual distress related to sexual activity in LGB + (i.e., lesbian, gay, bisexual, or other minoritized sexual orientations) individuals (Manão et al., 2023) and identified that this population faces specific distressing sexual problems—stemming from contextual and interpersonal factors—that go beyond the current criteria for ICD-11 (World Health Organization, 2019) and DSM-5 (American Psychiatric Association, 2022). In our current study, we aim to broaden the understanding of this field and expand the current knowledge by taking an experience-based approach by thoroughly examining how cis heterosexual people who self-report having distressing sexual problems during partnered face-to-face sexual activity describe this experience. The main research question we seek to answer is: “How is distressful partnered face-to-face sexual activity described by those who experience it?”. Complementarily, we aim to compare the answers of those who had and do not have sought professional clinical help. We will outline the reasons why our participants did not seek professional help and expand the knowledge derived from existing studies worldwide. We also intend to explore how experiences of partnered sexual distress may differ across genders. We intend to utilize an online qualitative methodology to gather data from diverse experiences, in line with Epstein’s (2023) argument that knowledge from non-experts may contribute to broadening and informing practices of citizenship, advocacy, and, in our view, healthcare practices. This approach is particularly valuable in fields such as sexology (Braun and Clarke, 2020), where qualitative studies can provide insights that enhance clinical practice and improve healthcare (e.g., Paulsen et al., 2023; Raposo et al., 2024) for individuals experiencing sexual distress in partnered face-to-face sexual activity.

## 2 Materials and methods

### 2.1 Participants

The study included 438 heterosexual participants (*M*_age_ = 41.1, *SD* = 12.2) who self-identified as having distressing sexual problems during partnered face-to-face sexual activity, of which 306 self-identified as ciswomen (69.9%) and 132 as cismen (30.1%). Regarding the relational configuration, 331 were in a monogamous relationship (75.6%), 56 stated that they did not have significant/s relationship/s (12.8%), 29 were having casual relationships (6.6%), 14 were in “Other not mentioned” (3.2%), and 8 were in a non-monogamous relationship (1.8%). Regarding nationality, 422 participants were Portuguese (95.13%) 12 were Brazilian (2.73%), 1 participant was Argentinian (0.23%), 1 was Cabo Verdian (0.23%), 1 was Finnish (0.23%), 1 was from EUA (0.23%), and 1 stated “I prefer not to say” (0.23%).

Of all participants, 157 identified as Catholic (37.7%), 135 declared themselves Atheist (32.5%), 28 indicated that they identified with “another option” (6.7%), 26 opted to state that they preferred not to answer, 63 identified as Agnostic (15.1%), 5 as Protestant (1.2%), 1 as Orthodox Christian (0.2%), and 1 as Hindu (0.2%).

Concerning the participants’ educational level, our data indicates that 169 individuals have attained an university degree (40.6%), 130 have pursued postgraduate studies (31.3%), 75 have completed secondary education (18%), 32 have participated in technical or vocational training courses (7.7%), 8 have attained the ninth grade (1.9%), 1 has not reached the ninth grade (0.2%), and 1 participant did not respond to this question (0.2%).

### 2.2 Dataset generation

The current study is part of a larger cross-sectional, online, mixed-methods project on sexual distress. It employed participatory design techniques (Cornwall and Jewkes, 1995) and involved collaboration with MUSEX – the Pedagogical Museum for Sex, Associação Gerador, and sex therapists certified by the Portuguese Society of Clinical Sexology (SPSC). These sex therapists provided expertise by reviewing the project’s content to ensure it was relevant, inclusive, and appropriately concise. Additionally, we sought feedback from laypeople, including LGBTQIA+ people, to review the inclusive language used in the project. We chose online recruitment by mutual agreement to expand our geographic reach and make participation more accessible and less time-consuming (Terry and Braun, 2017). After appreciation and approval by from all people involved, the study was disseminated online on different social media (e.g., Instagram, LinkedIn) through the research team’s professional accounts, by different groups in social media and through a collaboration with (MUSEX - the Pedagogical Museum for Sex, Associação Gerador, the Master’s Degree in Sexology at Universidade Lusófona, and SPSC), which also advertised the study on their social media. Following the advice of Braun et al. (2020), both the qualitative and quantitative content of the questionnaire were tested with individuals from the general population to confirm its clarity and comprehensibility before advertising the study. Using a snowball-like sampling technique, participants were also invited to share the questionnaire with others. Due to these recruitment strategies, we do not know how many people the study reached. We followed the qualitative research guidelines, recognising that it is problematic to determine *a priori* an ideal number of participants. Consequently, we did not prioritise a specific participant composition but focused on obtaining informative answers (Sim et al., 2018). Data was generated between March 9, 2023, and October 21, 2024.

In the larger cross-sectional, online, mixed-methods project, when accessing the study’s URL, participants were asked to read the informed consent in the first part of the survey and then provide their agreement or disagreement regarding participation. The second part of the survey involved answering a sociodemographic questionnaire. The third and final part of the survey had quantitative and qualitative questions. For the current study, we selected people who met two inclusion criteria: (1) self-identification as a heterosexual cisgender person and (2) self-reporting as having one or more distressing sexual problems during face-to-face sexual activity. We defined sexual activity as “sexual activity refers to mutual stimulation of genitals, oral sex, anal sex, intercourse, and other forms of face-to-face sexual stimulation” (Dove and Wiederman, 2000). We asked people about their help-seeking behavior in the context of their distress related to face-to-face sexual activity. “Our aim was to understand the context of distressing sexual problems related to partnered face-to-face sexual activity of heterosexual people, so we presented the following open question that we analyzed: Can you please describe your distressful experience with partnered face-to-face sexual activity?”.

The Ethical and Deontological Committee of the Ethical and Deontological Committee for Scientific Research of the School of Psychology and Life Sciences (CEDIC) of Lusófona University in Lisbon approved the study. All the ethical and deontological guidelines were followed, namely, the Helsinki Declaration and the European Textbook on Ethics in Research (European Commission: Directorate-General for Research and Innovation, 2010). All IP and geolocation information was deleted, and only the research team has access to the database, which is password protected. The informed consent indicated that the data set would not be shared and provided additional details about the study (e.g., aim, duration, no financial compensation). At both the beginning and end of the questionnaire, the contact details of the principal researcher were provided, along with a list of contacts for clinical and sexual support, should participants feel it would be beneficial to access them.

### 2.3 Reflexive statement

The following reflexive statement follows Braun & Clarke’s guidelines (Braun and Clarke, 2024) and refers to the people involved in the reflexive thematic analysis and the roles and activities they were performing during this analytical process.

Patrícia M. Pascoal is a cisgender Portuguese woman. She holds a European PhD in Clinical Psychology, teaches undergraduate students, and supervises postgraduate students pursuing master’s and doctoral degrees in medicine, psychology and sexology using qualitative and quantitative research methods. She also has experience in clinical practice in psychology and sexology as a cognitive-behavioral therapist (CBT) and CBT supervisor. She is involved in the executive boards and committees of diverse national (e.g., Portuguese Association for Behavioral Therapy) and international scientific associations (e.g., European Federation of Sexology; International Society for Sexual Medicine).

Andreia A. Manão is a cisgender woman from Portugal. She is pursuing her PhD in Clinical Psychology (CBT), focusing on both quantitative and qualitative research methods, while also receiving clinical training to become a clinical psychologist and sex therapist. Additionally, she teaches courses in the Bachelor’s program in Psychology and the Master’s programme in Sexology.

Catarina F. Raposo is a cisgender woman and a PhD candidate in human sexuality. She has experience in clinical practice within psychology and sexology and is currently training to become a clinical CBT therapist. She is a non-tenured invited lecturer, teaching mental health and sexology-related topics. Her role also includes supervising students pursuing master’s degrees in psychology and sexology during their internships.

### 2.4 Data analysis

We used SPSS 27 (IBM SPSS) for descriptive statistics (e.g., average age and standard deviation) and Microsoft Word for the reflexive thematic analysis.

The answers about having had/sought professional help were subjected to summative content analysis (Hsieh and Shannon, 2005) and were consolidated with the procedures for qualitative coding (Zhang and Wildmuth, 2009).

Regarding the qualitative answers to distressing sexual problems related to face-to-face sexual activity, we analyzed the data using reflexive thematic analysis to understand meaningful patterns in participants’ responses (Braun and Clarke, 2021). We adopted a contextual perspective, acknowledging that experiences are subjective (Braun and Clarke, 2022), meaning that the analysis occurs at the intersection of the researchers, the data and broader contexts. Our analysis was mainly inductive but also shaped by our knowledge and experience in psychology and/or sexual health content and related issues, making it, in this sense, also deductive. We followed the guidelines of reflexive thematic analysis (Braun and Clarke, 2021), which involved familiarizing ourselves with the data as it was submitted to the Qualtrics platform (Provo, UT, United States)—i.e., an online, secure survey platform. We then copied the data into a Word document and began to code it at both a surface and a deeper level, that is, in a semantic and latent way, respectively. This process was performed independently and simultaneously by three researchers, Patrícia M. Pascoal, Andreia A. Manão, and Catarina F. Raposo. Subsequently, the analysis was compared and refined collaboratively through reflexive discussion until we achieved a cohesive understanding of the data. Different opinions were encouraged to improve our data analysis and discussion rather than to achieve consensus. Comparisons across genders regarding experiences of sexual distress during partnered face-to-face sexual activity were conducted and discussed. Of the 438 participants, 22 offered unclear responses about the distressing partnered face-to-face sexual activity (e.g., *it’s tough*). To ensure our analysis accurately represents participants’ views, we excluded these 22 responses from our reflexive thematic analysis. Answers about self-reported excessive sexual interest were not analysed (e.g., *Every 16 seconds, I think about sex*; 58 years, man, without professional help) as they are not related to distress related to partnered face-to-face sexual activity. Thus, we excluded more 10 answers from our reflexive thematic analysis. Consequently, we analyzed 406 responses related to the question concerning distressing partnered face-to-face sexual activity. When reporting answers, in the “Results” section, minor adjustments have been made to some sentences to enhance their clarity. However, it is essential to emphasise that the meaning of these sentences has been totally preserved.

According to Braun and Clarke (2024), it is not recommended to split the “Results” and the “Discussion” sections in papers with reflexive thematic analysis (2024). However, considering the journal’s template, the authors decided it was adequate to follow the journal’s guidelines and therefore, we will separate the “Results” from the “Discussion” section, a possibility acknowledged by Braun and Clarke (2021).

In the upcoming sections, themes will be emphasized in **bold**, subthemes will be underlined, and quotes will be *italicized*. Every quote example includes concise sociodemographic details about the participants to protect their anonymity, such as age, gender, and the status of their experience with professional clinical help related to sexual problems (indicated as “with professional help” for those who have professional clinical help, “without professional help” for those who have not, and “did not indicate professional help status” for those who did not provide an answer about it).

## 3 Results

We will first present the results that describe the context and reasons for people who had and do not have sought professional clinical help, and then we will proceed with the reflexive thematic analysis results.

### 3.1 Professional help

Considering whether the participants had sought professional clinical help, our analysis revealed that 237 participants (54.1%) had not yet attended but expressed a desire to do so. Meanwhile, 47 participants (10.7%) had already attended clinical sexology/sexual medicine sessions, 59 (13.5%) were attending at the moment of data collection, and 58 (13.2%) expressed a desire to attend but were unable to do so.

Table 1 describes the reasons for not seeking professional clinical help. Of the 58 participants who expressed a desire to attend but were unable to do so, 21 (36.2%) did not answer this question.

**Table 1:** Frequency of code regarding the reasons for being unable to attend clinical consultations.

Codes (*n*)	Excerpts examples
Financial constraints (15)	*I have financial difficulties* (32 years, woman, without professional help)
Sexual problems as secondary issues (5)	*When I invest in myself it is in my mind, through psychotherapy appointments to resolve my traumas or major obstacles in life, putting sexuality to the other side, trying not to give it that much importance* (21 years, woman, without professional help)
Distress as not serious enough (3)	*I do not know if these are clinical issues* (33 years, woman, without professional help)
Shame related to seeking for professional help (3)	*I do not feel comfortable talking about sexual problems with anyone* (44 years, woman, without professional help)
Partner not being interested in having clinical consultations (3)	*My wife does not want me to seek help* (43 years, man, without professional help)
Self-management (2)	*I will solve it myself with practice.* (29 years, woman, without professional help)
Lack of confidence in the effectiveness of clinical consultations (2)	*I do not think help would work* (53 years old, woman, without professional help)
Time constrains (2)	*Unavailability of time* (34 years, woman, without professional help)
Do not know where to seek help (2)	*I do not really know where to look for help* (38 years, woman, without professional help)
Not wanting to discuss the problems with their partner (1)	*I did not tell my partner about the problem* (49 years, woman, without professional help)
Health professionals downplaying their problems (1)	*Gynaecologists relativise my problem, they associate my complaints only with daily stress* (43 years, woman, without professional help)
Alternative therapies (1)	*I have recently started using natural therapies* (50 years, woman, without professional help)
Absence of a partner (1)	*I do not have a partner* (36 years old, man, without professional help)
Not having a specialist available (1)	*For logistical reasons, I still have not managed to find the best help. My specialist doctor retired during the COVID lockdown and I did not get a new appointment* (57 years old, woman)

### 3.2 Reflexive thematic analysis

The length of the answers varied, from short answers (i.e., two or three words; e.g., *I have impotence*; 68 years, man, with professional help) to rich and descriptive answers (i.e., several sentences and rich content, for example, *I have a history of depression, and I go to therapy regularly. If I do not, the likelihood of having pain during sex again is significant. Therapy helps me to have emotional regulation tools so that this does not happen*; 29 years, woman, with professional help).

The themes are globally interrelated, as answers aggregate contents from several themes and subthemes (e.g., *My ex-boyfriend experienced premature ejaculation and erectile dysfunction. These issues contributed to the end of our relationship because he was unwilling to seek help and tell me about it. He even went as far as to take viagra without telling me. I only found out about this after we had not been sexually active for a while, and he began to feel unwell due to the increased heart rate caused by the stimulant*) (Age not disclosed, woman, did not indicate professional help status).

In terms of seeking professional clinical help, our analysis revealed a pattern of help-seeking behavior among people reporting experiences of pain during sexual activity, sexual dysfunctions (e.g., premature ejaculation), and physical medical conditions (e.g., haemorrhoids). Conversely, the answer of people experiencing sexual problems characterized by partner dynamics (e.g., poor communication, differences in sexual desire between partners) seems to be associated with lesser inclination to seek professional help.

We will now describe each theme, its corresponding subtheme/s, and provide detailed information with examples in the text and in Table 2.

**Table 2:** Results from the reflexive thematic analysis.

Themes	Subthemes	Example quotes
**Sexual (dys)function (It’s the function)**	-	*I have a history of depression, and I attend counseling regularly. If I skip these sessions, the likelihood of experiencing pain during sexual intercourse increases significantly. Therapy provides me with tools for emotional regulation, which helps prevent this pain during sex from occurring* (29 years, woman, with professional help)
		*I have lack of pleasure, cannot achieve orgasm and sometimes ejaculate prematurely* (36 years, man, without professional help)
		*I have erectile dysfunction* (79 years, man, with professional help)
**Intimacy dynamics (It’s us!)**	Impaired communication	*Difficulty communicating with partner* (24 years, woman, did not indicate professional help status)
	Discrepancies between partners	*My husband has a thing for erotic asphyxiation and I go along with it to give him pleasure. However, I must admit that I am finding it increasingly disagreeable* (32 years, woman, did not indicate professional help status).
		*My partner’s unwillingness to have sex with me* (39 years, man, without professional help)
		*Compared to me, my wife has a low sex drive* (51 years old, man, without professional help)
**Intrapersonal struggles (It’s me!)**	Physical health constraints	*Pain experienced in some situations, with and without penetration, related to endometriosis* (35 years, woman, with professional help)
		*I have gynaecological health problems* (41 years, woman, with professional help)
		*I develop vaginal/urinary infections very often, and that interferes with my sexual activity* (39 years, woman, with professional help)
		*I have hemorrhoids* (23 years, woman, did not indicate professional help status)
	Negative emotional response	*I need to shift my attention to my own performance. Because of that I find it challenging to let my body move in a more organic way that syncs with my emotions* (did not indicate age, man, did not indicate professional help status)
		*I’m afraid of having pain* (59 years, woman, did not indicate professional help status)
		*I have performance anxiety during sexual activity* (43 years, man, with professional help)
	Psychologic risk factors	*I usually do not feel aroused because I have negative feelings about my body* (50 years, woman, without professional help)
		*I have inhibitions during sexual activity, and I often fell shame* (33 years, woman, without professional help)
		*I experience excessive anxiety due to the fear of becoming pregnant* (22 years, woman, without professional help)
		*Shame causes me to overthink everything during sex* (45 years, woman, without professional help)
		*Some shame and discomfort because I am constantly too self-conscious about how my body might look in the eyes of any other person, whether it is attractive or not* (27 years, woman, did not indicate professional help status)
	Sociocognitive factors	*Growing up in the catholic religion influenced my views on sex, leading me to perceive it as something dirty. This also affected my thoughts on masturbation and pleasure* (45 years, woman, without professional help)
		*Inhibition, shame, [because] I have limiting beliefs about sexual activity* (33 years old, woman, without professional help)

The theme **Sexual (dys)function (It’s the function)** centers on distressing problems related to face-to-face sexual activity involving the genitals. These problems are typically described in terms that align with diagnostic criteria for sexual dysfunction, including erectile difficulties, premature ejaculation, challenges or inability to reach orgasm, lack of pleasure during sexual activity, pain during penetration, vaginismus, vulvodynia, and bleeding during vaginal penetration. This theme represents a key conceptual thread in the data and does not include subthemes.

The theme of **Intimacy dynamics (it’s us!)** includes distressing experiences grounded in a relationship. Its subthemes are Impaired Communication, which pertains to difficulties in self-disclosing oneself to the partner, and Discrepancies between partners, which describe problems in managing discrepancies related to sexuality, for example, sexual preferences and levels of sexual desire.

Finally, the theme of **Intrapersonal struggles (it’s me!)** aggregates descriptions of sexual distress with partnered face-to-face sexual activity that are related to individual characteristics, physical or psychological, of the respondent. A relationship may accentuate or diminish these characteristics, but they are not relationship-dependent. Its subthemes are: Physical health constraints that aggregates distress related to organic conditions that make sexual activity difficult (e.g., vaginal dryness, endometriosis, chronic pelvic pain, side effects of medications that affect sexual function); Negative emotional response a subtheme that pertains to negative emotional experiences during sexual activity (e.g., performance anxiety; fear); Psychological risk factors a subtheme that includes existing psychological traits that are risk factors for psychopathology and for psychological distress and that interfere with a complete experience of sexual activity (e.g., excessive anxiety/worry/fear, neuroticism, inhibition, feelings of inadequacy regarding one’s body); and Sociocognitive factors a subtheme specifically about negative attitudes towards sex and rigid beliefs derived from one’s education and socialization regarding what sexual activity should be (e.g., sex as dirty and immoral) that accentuate the distressful experience.

Concerning the participants’ gender, our analysis revealed distinct patterns. Among women, the predominant pattern was characterised by a lack of sexual desire, experiences of sexual pain, difficulties in achieving orgasms, and inadequate lubrication. In contrast, men exhibited a pattern of answers that emphasised premature ejaculation, erectile dysfunction, and a lack of interest from their partners in being involved in sexual activity.

Concerning the patterns of the content of the answers between people who had sought or not sought professional clinical help, our However, our study adds more layers to current formulations by emphasizing the professional clinical help present answers where relevance is placed on genital function together with a negative emotional experience, namely intense negative emotions related to sexual pain.

## 4 Discussion

This study analyzes self-reported experiences of people who self-identify has having sexual distress in partnered face-to-face sexual activity. It uses summative content analysis to describe and quantify their patterns of help-seeking. It has as a main goal to explore how people who self-report experiencing one or more sexual problems describe their sexual distress during face-to-face partnered sexual activities, utilizing reflexive thematic analysis to examine their responses. We will discuss the patterns of help-seeking behavior, the results of the reflexive thematic analysis, and the comparison across the answers across genders and between those who seek and do not seek professional.

A central theme that we developed, **Sexual (dys)function (It’s the function)**, relates to the impairment of sexual distress response, a crucial aspect for the diagnosis of sexual dysfunction supporting current diagnosis manuals [ICD-11 (World Health Organization, 2019), DSM-5 (American Psychiatric Association, 2022)] and supporting that problems with sexual response are an essential precursor for sexual distress. However, our study adds more layers to current formulations by emphasizing the sexual distress related to the absence of pleasure. Sexual pleasure has gained attention due to its central role as a sexual right (World Association for Sexual Health, 2021), and its study has received immense attention in the last years, resulting in a comprehensive proposal for its understanding (Laan et al., 2021). Despite this interest, currently, sexual pleasure is modestly mentioned in category-based diagnosis manuals, and, except for its mention in sexual interest arousal problems, there is no solid and clear role for sexual pleasure as an central concept or a criterion that contributes to the diagnosis of a sexual dysfunction or problem. Taken together, the results of the current study and existing research on sexual pleasure (Laan et al., 2021; Manão et al., 2023; Pascoal et al., 2020) accentuate the need to integrate sexual pleasure as a fundamental factor in the assessment, intervention, and clinical research of distressful sexual problems related to partnered face-to-face sexual activity.

The theme named **Intimacy dynamics (It’s us!)** is in line with previous research that reinforces that relationship factors are fundamental for the experience of sexual distress (Martins et al., 2024). Our results identify the relationship elements that operate during partnered face-to-face activity, namely sexual communication and the existence of discrepancies among sexual preferences. Depending on the type of relationship, it is well known that both expressive and instrumental communication (MacNeil and Byers, 2009) are fundamental for positive outcomes, for example, sexual satisfaction (Frederick et al., 2016). However, most research about communication during sexual activity focuses on the role that “sexual talk” or “dirty talk” has as a maximizer of sexual arousal and promoting orgasm (Frederick et al., 2018). Our results complement this data by highlighting that inhibition to speak and challenges with sexual self-disclosure during sexual activity are also characteristics of during partnered face-to-face sexual activity. This implies that communication during sexual activity follows a dual path: while certain types of communication may enhance arousal and satisfaction, inhibition and difficulties in communicating may intensify the experience of distress. The latest may be particularly detrimental when difficulties in communicating with a partner about sexual pain lead to painful, distressing partnered sexual activity (Oesterling et al., 2025). Furthermore, our results also support the notion that discrepancies in sexual preferences (e.g., levels of desire or arousal for certain emotions or practices) (Dewitte et al., 2020; Cardoso et al., 2023) are characteristic of sexual distress during sexual activity. There is an interrelation between these two subthemes, as the ability to express preferences and communicate about discrepancies is important to overcome the negative impact that these discrepancies may have. Logically, those whose communication is compromised due to relational factors may have a heightened experience of distressing partnered sexual activity. Additionally, recognizing this discrepancy and the challenges in sexual communication may lead individuals to become cognitively distracted during sexual activity. This distraction arises from an increased focus on communication difficulties during sexual activity, which can undermine arousal and pleasure (Tavares et al., 2020). Our results highlight the role that communication behaviors and sexual self-disclosure skills (Döring and Byers, 2024) have in minimizing sexual distress with partnered sexual activity.

In the theme named **Intrapersonal struggles (it’s me!)**, a puzzle of individual determinants is presented. If the experience of bodily-related difficulties, primarily due to illness or medication, is not a novelty as a determinant of distress (e.g., Zanolari et al., 2023), the participant’s descriptions accentuate that this sexual distress is marked and defined by the intense experience of negative emotions, namely experiences of anxiety and anguish. These descriptions are in line with research that places sexual dysfunctions and problems with partnered sexual activity as clinically meaningful experiences that can be approached clinically from a dimensional perspective within the spectrum of internalised disorders (Forbes et al., 2016). It is possible that these negative emotional experiences may interfere with the standard processing of arousal cues, particularly in assessing them. The vulnerability psychological factors developed in subtheme Psychologic risk factors (e.g., body awareness) seem to be traits or steady psychological characteristics that may be triggered or maximised during sexual activity. They may compromise the total immersion in the erotic experience, namely, by promoting cognitive distraction (Barlow, 2016), for example, distraction with one’s body appearance (Carvalheira et al., 2017; Dove and Wiederman, 2000; Manão and Pascoal, 2023; Pascoal et al., 2019). These steady cognitive characteristics and psychological traits frame cognitive processing during sexual activity, which is consistent with cognitive models of sexual dysfunction that defend that cognitive processes define and shape the erotic experience and are explanatory processes that define the experience of sexual dysfunction. Our results highlight that these processes also shape the experience of distress, accentuating it and aligning it with cognitive models of sexual dysfunction. Sociocognitive factors is a subtheme that is linked to socialization, encompassing negative messages that have been internalised about sexuality, which further compromise the sexual experience. These factors, commonly referred to as sexual beliefs, are significant components of cognitive models of sexual response (Nobre, 2023). These sexual beliefs, shared by men and women, tend to be rigid (Pascoal et al., 2018) and compromise complete immersion and openness to experience (Peixoto and Nobre, 2017). They play a crucial role as predictors of negative sexual experiences as they disrupt attentional focus during sexual activity by promoting ongoing surveillance of the experience in accordance with these beliefs, potentially undermining the whole experience of satisfactory arousal. In the current study, it is emphasised that social norms and beliefs may also impact the experience of distress.

The current results refine the clinical meaning of sexual distress with partnered face-to-face sexual activity, differentiating it from sexual distress as a global concept and from psychological distress and describing its unique features. It has important implications for theory building, sex therapy/clinical intervention, and clinical research. The study highlights that sexual distress with partnered face-to-face sexual activity is a detrimental sexual outcome associated with strong negative emotions essentially related to sexual response, personal physical difficulties (e.g., illness) and psychological factors (e.g., shame, body dissatisfaction) that promote cognitive distraction during sexual activity, problems with partnered communication about sexual preferences and discrepancies and negative attitudes and rigid beliefs about sexual activity. These factors point to the direction of a comprehensive theoretical model of sexual distress related to sexual activity that includes SDRSF and conceals conceals both cognitive (Barlow, 2016; Nobre and Barlow, 2023) as well as relationship factors (Martins et al., 2024). We propose that, following a top-down approach, a flexible, integrative, and comprehensive theoretical model of distress with partnered face-to-face partnered sexual activity should integrate SDRSF features namely: the experience of some level of impairment and negative emotions related to sexual function and response, including difficulties in experiencing pleasure and satisfaction; discrepancies between partner desires and preferences as well as difficulties in expressing and communicating about sexuality with partners; and both non-sexual related (body dissatisfaction, inhibition, neuroticism) and sexual-related (rigid negative beliefs) personal characteristics, some of which are derived from broader cultural contexts (e.g., family, religion). Integrating our findings and overcoming the limitations of this and previous research will contribute to establishing such an endeavour.

In terms of implications for sex therapy/clinical intervention, the current study calls for attention to the need to develop integrative approaches that address negative emotions (e.g., anxiety) that we found associated with sexual distress with partnered face-to-face sexual activity. These emotions are often maintained by latent psychological factors such as neuroticism and may benefit from transdiagnostic approaches to emotional problems (Sauer-Zavala et al., 2017) Interventions should include cognitive processes, such as CBT (Brotto et al., 2025) particularly those tailored to sexual dysfunction in the context of illness (Pieramico et al., 2024). Additionally, couple-focused and systemic interventions that target relationship dynamics, particularly fostering positive and respectful communication about sexual discrepancies, are also recommended (Davies et al., 2021). This raises important issues related to the education of sexual health professionals, namely sex therapists, reinforcing that addressing sexual dysfunctions requires a solid training background that needs to be combined with deep knowledge of relevant, empirically based theoretical models of intervention to ground best standards for clinical practice. Furthermore, sex therapists should complement it with professional updates in relevant techniques. The current results therefore support that professional education in sex therapy should be grounded in pre-existing formal training in relevant psychotherapies and should include content related to mental health, psychopathology, CBT approaches, and integrative and systemic models of intervention. Regarding research, the most significant impact of the current study pertains to the development of randomized controlled trials (RCTs) to intervene in sexual dysfunction or partnered face-to-face sexual activity related problems, as these need to be integrative and focus on a “an adequate” outcome measure of sexual distress. This measure should address sexual response issues while also being sensitive to therapeutic change, such as the SFEQ (Mitchell et al., 2022), which aligns more with our current findings. However, it also needs to incorporate secondary outcome measures, including sexual pleasure (Manão et al., 2023; Martins et al., 2024) and assessments of negative emotional experiences, such as anxiety and depression (Soler et al., 2021). Furthermore, future interventions should target processes known to influence negative emotional states (e.g., repetitive negative thinking) and the features and maintaining processes of sexual distress with partnered sexual activity that can be altered through therapeutic means (e.g., cognitive distraction, communication skills, sexual beliefs/psychological flexibility). It is important to note that the existing RCTs are aimed at specific clinical conditions (e.g., Banbury et al., 2021; Brotto et al., 2021) and that the results of this study support the possibility of crossdiagnostic RCTs, i.e., RCTS aimed at people with different diagnoses of sexual dysfunctions. At the same time, it is also highlighted that gender differences may be considered in designing such RCTs as women and men participants in crossdiagnostic interventions may differ in their complaints.

As our results highlight, many reasons that prevent people from seeking professional clinical help are related to accessing professional sexual healthcare (such as shame about sexual issues, cost, and geographic distance), we propose that developing internet interventions (whether blended or not) may be a practical solution for delivering pilot studies that incorporate such integrative transdiagnostic-based approaches. Regarding the reasons for not seeking professional clinical help, our results are in line with research in the field, revealing that most people who experience a sexual problem related to partnered face-to-face sexual activity do not seek professional help (e.g., Lafortune et al., 2023; Moreira et al., 2005), namely due to time and financial constraints.

Furthermore, this research reveals that institutional and professional trust-related factors prevent people from seeking help. It would be important to better understand if these factors are related to reports of bad experiences or just a lack of knowledge about the ongoing education and certification that exists in the field of clinical sexology and sex therapy and/or about existing specialized services that exist in Portugal (Alarcão et al., 2017; Raposo et al., 2024). The lack of confidence in specialized services and the pursuit of alternative approaches render people who suffer from sexual problems vulnerable to the practice of non-evidence-based practices, a problem that could only be overcome with higher levels of health literacy (Liu et al., 2020).

It is worth highlighting that partners’ compliance is also a significant obstacle that may be linked to a dominant representation of sexual problems as an individual problem and not a systemic, relationship-embedded issue. This position may reflect a view based on healthism, which tends to overemphasize a person’s role in determining their own health outcomes, ignoring the contexts in which these outcomes are shaped and embedded (Crawford, 1980).

Lastly, viewing sexual problems as a minor concern may explain why some people do not seek help, as they might not experience significant distress during partnered sexual activity. This has also been suggested by Hendrickx et al. (2016a, 2016b). Overall, the results reveal that there are people who are distressed by their sexual activity and are not accessing healthcare services due to different obstacles and that these should be approached through better education regarding the available services and professionals who are licensed and prepared to act on sexual problems. Moreover, these people, independent of the severity of the symptoms and compatibility with a formal diagnosis of sexual dysfunction, may benefit from internet-delivered treatment options. These solutions not only demonstrate positive results for their specific outcomes of interest (e.g., sexual function; Zarski et al., 2022) but also are a solution to barriers to treatment, such as improving time management, reducing stigma of asking help/having a sexual problem, reducing financial limitations and geographic barriers (Costa et al., 2023; Rodda and Luoto, 2023). Furthermore, these options likely address broader systemic problems, including social inequalities and deficiencies in health and sexual health follow-up care among minority groups, including people with physical disabilities, who do not have to face physical barriers to receiving treatment if the intervention is delivered online (Costa et al., 2023).

Finally, the comparison of content across genders is in line with research that demonstrates women experience more distressful sexual problems related to sexual function than men (Cobbs et al., 2022), which stresses that pursuing penetrative sex is more detrimental for women as they are more exposed to pain derived from penile-vaginal penetration. Because they prioritize their partners’ sexual enjoyment they are subsequently exposed to more distress related to sexual activity. The findings may be influenced by the sample composition, which was predominantly female (72.5%) and there could be a response bias, as men may experience sexual distress but might be unable or unwilling to disclose it. It could also be because men give more relevance to their spouse’s lack of sexual desire as a cause for their distress, and not to other problems their spouses may face, such as painful sex. It can be that men’s pressure to diminish the discrepancy between sexual desire results in more frequent non-desired sexual activity because women do not disclose their difficulties and, subsequently, have more painful sex. This possibility has been explored (Oesterling et al., 2025) and reveals that even in the presence of function problems, sexual distress is inbound to relationship factors.

Overall, our results have similarities with both the DSM-5 and ICD-11, indicating that sexual dysfunctions arise from a complex interplay of biological, psychological, and sociocultural factors. This is particularly evident in the theme **Sexual (dys)function (It’s the function)**, which highlights difficulties related to sexual function, and was the most prominent pattern of meaning developed. Our findings support the emphasis of these psychopathology manuals on considering a range of contributing factors when assessing sexual dysfunctions, including relationship dynamics, such as poor communication and discrepancies in desire, as illustrated in the theme **Dynamics of Intimacy (It’s us!)**. Interestingly, participants did not identify partner-related issues as significant sources of distress, possibly due to the self-referential nature of the online questionnaire used. Our results also emphasize individual vulnerability factors (e.g., negative body image), psychiatric comorbidities (e.g., depression and anxiety), and sociocultural influences (e.g., religious or moral beliefs about sexuality) that contribute to the theme **Intrapersonal Struggles (It’s me!)**. Nonetheless, our findings expand the psychopathology framework of the DSM-5 and ICD-11. While manuals focus on problems that meet clinical thresholds, participants reported distress related to sexual experiences that may not fit these criteria, yet remain significant. Issues such as feelings of inadequacy and unmet expectations can lead or be components of sexual distress. Unmet expectations, for example, may account for lack of sexual pleasure, which can be a critical factor not adequately addressed by DSM-5 and ICD-11 criteria. In other words, manuals do not explicitly include the subjective absence of sexual pleasure as a standalone clinical concern, nor explore its relationship to the experience of distress. Additionally, both DSM-5 and ICD-11 tend to underrepresent the impact of socioculturally shaped meanings and internalized scripts, which were central to the experiences of our participants. Therefore, our findings support and extend the ICD-11 framework by advocating for a more comprehensive understanding of sexual problems.

The current study presents several limitations that need to be considered to frame the results and their interpretation, as well as to inform future studies. Firstly, it is not possible to determine whether the participants are eligible to have a diagnosis of sexual dysfunction, which could further inform our analysis. They do self-report distress and problems during partnered sexual activity, but these may be explained by factors that were not properly addressed and evaluated (e.g., organic cause, the existence of severe psychopathology). Furthermore, we did not evaluate the sexual distress with partenered face-to-face sexual activity levels, which could have facilitated the determination of whether seeking professional clinical help is associated with the sexual problem/s themselves or the distress concomitantly experienced. Future research should aim to measure sexual distress levels using targeted measures (e.g., SFEQ). The study design, i.e., relying on straightforward answers that were not discussed or explored in-depth, which can happen during interviews, did not allow us to gain a more nuanced understanding of the context in which the experience of distress emerged. It can be that some people report distress related to sexual activity that emerges after the sexual encounter and that derives from a lack of pleasure, satisfaction, or shame. Also, the fact that we gave a definition of sexual activity that focused on specific practices may have primed participants to focus more on distress related to sexual function or intercourse. It is also important to note that some participants, predominantly men, whose answers were not included in the reflexive thematic analysis, reported that experiencing high levels of sexual interest or drive was a distressing sexual problem for them. Although this problem did not appear to be directly related to sexual activity, it warrants further investigation in future studies. This suggests that sexual distress related to partnered face-to-face sexual activity may persist after the sexual encounter and be the focus of concerns. This finding that was not analyzed, should be taken into account in future studies. Additionally, we could not determine whether the notable gender differences were related to the sexual double standard, which refers to the tendency to evaluate genders differently for identical sexual behaviors (Milhausen and Herold, 2008, Gómez-Berrocal et al., 2022), such as men enjoying greater sexual freedom while women’s sexuality is more taboo (Raposo et al., 2024). Research suggests that sexual responses experienced by women are associated with this standard, which negatively impacts sexual response (Álvarez-Muelas et al., 2022). This should be examined in future studies. Finally, it is vital to consider the sociocultural context of our predominantly Portuguese participants and their influence on results. To our knowledge, there are no existing studies specifically examining help-seeking for sexual problems within the Portuguese population. However, available evidence suggests that sexology in Portugal has gradually gained visibility and recognition (Alarcão, 2017). Moreover, a recent study (Manão et al., 2024) found that Portuguese individuals’ understanding of sexual health includes the notion of seeking support from healthcare services, suggesting a growing openness to professional help. However, this positive perception did not fully translate into behavior, as our findings showed that some of the participants reported experiencing sexual distress without seeking help, which is in accordance with other research about help-seeking behavior (e.g., Moreira et al., 2005; Moreira et al., 2008). Conducting similar studies in different cultural settings could help clarify whether these findings are context-dependent or reflect a more widespread phenomenon. Not only, but especially, we recommend that future studies on this topic be conducted outside of Western, Educated, Industrialized, Rich, and Democratic (WEIRD) populations, in order to fully capture the diversity of sexual distress with partnered face-to-face sexual activity. This recommendation aligns with the calls of scholars such as Klein et al. (2022a, 2022b) to decolonize science and promote greater contextual diversity in sex research.

## 5 Conclusion

The analysis of people’s definitions of sexual distress with partnered face-to-face sexual activity reveals its strong interrelation and overlap with SDRSF (with a strong focus on genital response and penile-vaginal penetrative sex), relationship factors, and specific individual factors. This result aligns with the current approach to sexual dysfunctions by both DSM and ICD, which emphasises the contextual nature of sexual dysfunctions and recognises both individual and interpersonal features in diagnosing sexual dysfunction. However, the current study innovates by offering a nuanced perspective on how sexual distress with partenered face-to-face sexual activity should be assessed and approached, demonstrating that some of the associated features (e.g., anxiety/worry; inhibition; lack of communication skills) are crucial components of this distress that must be targeted in intervention. Our results support an integrative approach to clinical intervention and research that combines a transdiagnostic approach with CBT-derived and systemic theoretical models of intervention, considering sexual distress with partenered face-to-face sexual activity or SDRSF as the primary outcome but that also includes sexual pleasure and psychopathology as necessary outcomes of clinical intervention.

## Data Availability

The datasets presented in this article are not readily available because the dataset can be shared upon reasonable request to the corresponding author. Only raw anonymized data will be shared. Requests to access the datasets should be directed to patricia.pascoal@ulusofona.pt.
